# Increased Ankle Plantar Flexor Stiffness Is Associated With Reduced Mechanical Response to Stretch in Adults With CP

**DOI:** 10.3389/fbioe.2021.604071

**Published:** 2021-03-25

**Authors:** Jakob Lorentzen, Rasmus Feld Frisk, Jens Bo Nielsen, Lee Barber

**Affiliations:** ^1^Department for Neuroscience, University of Copenhagen, Copenhagen, Denmark; ^2^Elsass Foundation, Charlottenlund, Denmark; ^3^School of Applied Health Sciences, Griffith University, Brisbane, QLD, Australia

**Keywords:** ankle stiffness, biomechanical evaluation, cerebral palsy, contractures, electrophysiology, spasticity

## Abstract

Hyperexcitable stretch reflexes are often not present despite of other signs of spasticity in people with brain lesion. Here we looked for evidence that increased resistance to length change of the plantar flexor muscle-fascicles may contribute to a reduction in the stretch reflex response in adults with cerebral palsy (CP). A total of 17 neurologically intact (NI) adults (mean age 36.1; 12 female) and 13 ambulant adults with CP (7 unilateral; mean age 33.1; 5 female) participated in the study. Subjects were seated in a chair with the examined foot attached to a foot plate, which could be moved by a computer-controlled electromotor. An ultrasound probe was placed over the medial aspect of the leg to measure the length of medial gastrocnemius muscle fascicles. Slow (7 deg/s) and fast (200 deg/s) stretches with amplitude 6 deg of the plantar flexors were applied over an ankle range of 70 deg at 10 deg intervals between 60 and 130 deg plantarflexion. It was checked by EMG electrodes that the slow stretches were sufficiently slow not to elicit any activity and that the fast stretches were sufficiently quick to elicit a maximal stretch reflex in both groups. The torque elicited by the stretches was measured together with changes in the length of medial gastrocnemius muscle fascicles. Muscle fascicles increased significantly in length with increasing dorsiflexion position in both populations (*p* < 0.001), but the fascicles were shorter in the CP population at all positions. Slow stretches elicited significantly larger torque and significantly smaller length change of muscle fascicles as the ankle joint position was moved more towards dorsiflexion in CP than in NI (*p* < 0.001). Fast stretches elicited larger torque responses at ankle joint positions of 80–100 deg in the NI than in the CP group (*p* < 0.01). A significant negative correlation was observed between the torque response and muscle fascicle length change to slow stretch in CP (*p* < 0.05), but not in NI. These findings support that increased passive resistance of the ankle plantar flexor muscle-tendon unit and development of contractures may conceal stretch reflex response in adults with CP. We argue that this should be taken into account in the neurological examination of spasticity.

## Introduction

Cerebral palsy (CP) is a congenital neurodevelopmental disorder caused by a non-progressive disturbance of the developing brain, which occurs before, at the time of or shortly after birth (Bax et al., 2005; Graham et al., 2016). The disabilities caused by the primary lesion and secondary problems such as contractures and joint deformities continue to be a challenge also in adulthood and recent observations suggest that significant further deterioration of functional abilities are common at relatively young age in adults with CP (Bottos et al., 2001; Morgan and McGinley, 2014; Cremer et al., 2017). Since CP is the most common cause of childhood disability and life expectancy is close to that of the background population, the population of adults with CP, who require health services and social support, is as large as for several other neurological disabilities in adults such as multiple sclerosis and Parkinson’s disease (Peterson et al., 2015). Despite of this, there is comparatively little research performed in adults with CP and we therefore have relatively little knowledge of the nature of their functional challenges and problems.

Recent studies using objective electrophysiological and biomechanical techniques have indicated that it is reduced central neural drive, reduced muscle mass and altered muscle mechanics, including increased passive (non-neural) stiffness leading to reduced joint mobility (contractures) and joint deformities, which are the main causes of limited functional capacity and reduced gait ability in the group of ambulant (high-functioning) adults with CP (Geertsen et al., 2015; Gillett et al., 2018; Yamaguchi et al., 2018; Frisk et al., 2019a,b). In contrast, hyperexcitability of stretch reflexes and muscle “over-activity” appear to have no or little significance (Geertsen et al., 2015; Frisk et al., 2017). However, the observation of lack of exaggerated stretch reflexes should be taken with some caution. Several factors influence the size of the stretch reflex when elicited under standardized conditions during a biomechanical and electrophysiological objective examination, but probably the most important is to which extent the stretch reaches the muscle spindles in the investigated muscles and causes a response of the muscle spindle afferents. In healthy muscles that are exercised regularly, muscle spindles respond quickly and effectively to even very small stretches, but muscles in persons with central motor lesions are different in a number of ways that may change this dramatically. When muscles are inactive, the extrafusal muscle fibres undergo atrophy (Bodine, 2013), which may in itself change the mechanical efficiency of a stretch in activating the muscle spindles, which are placed mainly in parallel with the extrafusal muscle fibres (Windhorst, 2008). Atrophy has been shown to alter the pennation angle of the muscle fascicles, which may result in an altered response of the muscle spindles (Narici et al., 2016). The muscle spindles themselves also undergo alternations in structure and composition of their membrane receptors, which may lead to altered responses to stretch (Carrasco et al., 2017). Importantly, the elastic elements in the muscle such as the connective tissue in the extracellular matrix proliferate and become stiffer, which may eventually result in reduced range of movement and manifest contractures (Singer et al., 2001; de Bruin et al., 2014; Jalal et al., 2020). These alterations in the muscles may be demonstrated within a few days after central motor lesions, but develop gradually over weeks, months and even years following the primary lesion – likely depending on complex genetic and environmental factors in the individual case (Jalal et al., 2020). Due to their early occurrence and their significant impact on functional ability, it has been suggested that these alterations in muscle architecture and function should be considered a muscle disease in its own right (Baude et al., 2019).

Clarification of the interaction between these changes in muscle properties and stretch reflex activity is important for directing clinical practice and treatment. Such an interdependence has indeed been found in children with CP (Bar-On et al., 2018).

In the present exploratory study we investigated whether altered muscle architecture and soft tissue resistance may reduce the ability of an applied stretch in reaching muscle spindles and elicit a mechanical response to stretch in adults with CP.

## Materials and Methods

A total of 13 adults with CP aged 33 ± 7years (5 females, 8 males; 6 GMFCS I, 3 GMFCS II, and 4 GMFCS III) and 17 neurological intact adults (NI) aged 36.1 ± 4.5years (11 females and 6 males) participated in the study. The CP participants were recruited from the Danish Cerebral Palsy organization. Adults diagnosed with spastic type CP were included if they were independent ambulant with/without walking aid (GMFCS I–III), and had reduced range of motion (ROM) in the ankles or increased passive stiffness in plantar flexors. Participants with CP were excluded if they had received lower limb intramuscular injection with Botulinum toxin type-A within 6 month. The TD participants were recruited from the local community to participate in this study, were matched for age and were required to have had no lower limb injury in the six months prior to testing. All participants were excluded if they had received lower limb orthopaedic surgery or injury within the previous two years. However, 11 of the 13 participants with CP had one or more Achilles tendon elongational surgery during their childhood

Three of the 13 participants with CP had been taking antispastic medication (Baclofen) on a daily basis for more than 5 years. However, all participants were asked to omit taking any anti spastic medication prior to the examination on the day of the study.

The study was approved by the local ethics committee (H-2-2014-028) and all procedures were conducted within the standards of the Helsinki declaration. Prior to experiments, all subjects received written and verbal information, and written consent for participation was obtained.

An overview of the participants is given in Table 1.

**TABLE 1:** Demographic information about all participant.

	Adults with CP *n* = 13	NI Adults *n* = 17
Age (Years)	33.1 (SD:10.9; range 23–56)	36.1 (SD:4.5; range 26–55)
Gender (% Female)	39	67
Weight (Kg)	63.5 (SD:7.0)	64.4 (SD:9.4)
Height (CM)	169.7 (9.2)	169.8 (8.7)
GMFCS (1-5)	1 = 6; 2 = 3; 3 = 4	
ROM (deg DF deficit)	0 = 3; −10 = 6; −15 = 2; −20 = 2	0 = 17
MAS (PF)	1 = 5; 2 = 5; 3 = 3	
Strength (PF)	2 = 1; 3 = 4; 4 = 6; 5 = 2	5 = 17
Achilles reflex (% hyperactive)	39	0

*The first row shows the participants age in Years and standard deviation (SD); the second row shows the representation (in %) of female among the participants; the third row shows the weight in Kg and SD; the fourth row shows the height in CM and (SD); the fifth row shows the participants General Motor Performance Classification Score (GMFCS; 1–5) (first the score and them the number of participants with this score); the sixth row shows the reduction in ankle dorsi flexion (DF) range of motion (ROM) where the number represents the reduction in dorsi flexion in degrees followed by the number of participants with this reduction; the seventh row shows the Modified Ashworth Score (MAS) in the plantar flexor muscles (PF) (first the score and then the number of participants with this score); the eight row shows the strength in plantar flexors measured by the Medical Research Council scale for muscle strength (MRC; 1–5) (first is indicated the score then the number of participants with this score); the nineth row shows the representation of participants with hyperactive Achilles reflex activity (in %).*

### Test Method

#### Passive and Reflex Torque

In order to objectively assess the passive and reflex mediated stiffness components of the ankle plantar flexors biomechanical and electrophysiological evaluation was performed according to previous methods (Lorentzen et al., 2010).

**FIGURE 1:**
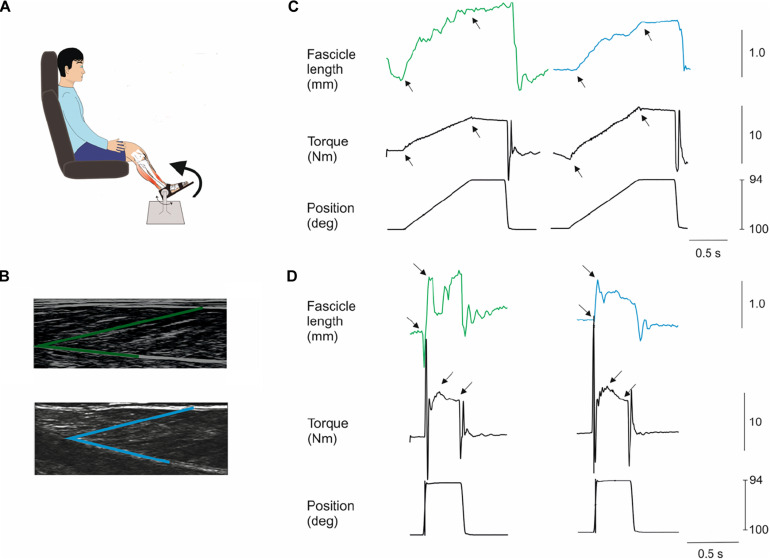
Methods. Subjects were seated in a reclining armchair with the examined leg supported and the foot attached to a plate which could be rotated by a computer-controlled electromotor **(A)**. The hip joint was positioned in 100 deg flexion and the knee fully extended (illustration not true to reality in this respect). Slow (7 deg/s) and fast (200 deg/s) dorsiflexion rotations of the foot plate were applied at different positions of the ankle joint throughout the range of movement at 10 deg. intervals. An ultrasound probe was placed over the medial aspect of the lower limb to monitor the length of medial gastrocnemius muscle fascicles during the stretches **(B)**. A single fascicle was selected (marked in green for an NI individual and in blue for an individual with CP) and its length from the superficial to the deep aponeurosis of the muscle was tracked throughout the experiment. **(C,D)** show examples from an NI individual (left hand graphs) and individual with CP (right hand graphs) of the movement of a fascicle and the torque on the foot plate (black) during a slow **(C)** and a fast stretch **(D)** at 100 deg initial ankle position. Black arrows indicate the interval for measurement of fascicle and torque amplitudes. Note the peak in the torque measurement following the fast stretch in **(D)** indicated by the first arrow. This is the increase of torque elicited by the stretch reflex. The amplitude of this peak was measured in relation to the baseline torque during the holding phase of the stretch as indicated by the second arrow in the torque graphs of **(D)**. All fascicle measurements are in mm, all torque measurements are in Nm and position are in deg.

Briefly, subjects were seated in a stable reclining armchair that was fixated to the floor with the knee joint secured in a stretched position. The examined foot was attached to a footplate, which could be rotated by a motor (CEM model 26) (Figure 1A). The medial malleolus was aligned to the rotational center of the device and the ankle position was secured throughout the experiment by two straps. One strap around the proximal part of the foot and the pedal securing that the heel kept contact to the footplate and one strap around the distal part of the foot and the foot plate securing the distal contact between the distal part of the foot and the footplate at all times throughout the experiment. The motor was driven by a DC power amplifier (Brüel&Kjaer; model 2708) and could deliver maintained torques up to 80 Nm and peak torques up to 120 Nm. An electro-goniometer, connected to the foot plate, measured the foot plate angle and a torque meter measured the torque exerted on the foot plate prior to and during the stretch perturbations. The initial position of the talocrural joint angle was set to 90 deg which was measured with a manual goniometer and subsequent positions were adjusted according to this position. The hip joint was positioned in 100 deg flexion and the knee fully extended. The position of the knee was secured by fixation of the lower part of the thigh with 20 cm broad Velcro straps fixed to the floor.

Perturbations of the ankle were made in eight different initial ankle positions in 10 deg increments between 60 and 130 deg plantar flexion. The perturbations consisted of ramp and hold dorsiflexions with an amplitude of 6 deg at two velocities, 7 deg/s (slow; rise time 900 ms) and 200 deg/s (fast; rise time 10 ms) and with a hold time of 460 ms and fall time of 40 ms. The perturbation characteristics were similar for the two groups of participants. However, due to limitations in ankle ROM in some of the participant not all initial stretch positions were possible in all participants. All participants achieved ankle angles from 80 to 120 deg. A total of 130 and 60 deg positions were possible in 15 NI participants but no participants with CP. A total of 70 deg position was possible in all NI participants but only in seven participants with CP. Ten perturbations per velocity were delivered alternately starting with a slow stretch with a 2 s interval between each perturbation. This procedure was used for all positions.

EMG recordings were made in order to ensure that no muscle activity occurred during slow stretches and that stretch reflexes were elicited during fast stretches. EMG activity was recorded using bipolar electrodes (Ambu Blue sensor N-10-A/25, Ambu A/S Ballerup. Recording area 0.5 cm^2^ inter-electrode distance, 2 cm) placed over the soleus muscle. Recordings were made from the soleus muscle in order not to interfere with ultrasound measurements (see below) and since stretch reflexes have a lower threshold and larger amplitude in this muscle as compared to the other plantar flexor muscles. The skin was brushed softly with sandpaper (3M red dot; 3M, Glostrup, Denmark). A ground electrode was placed on the distal part of the tibia. EMG signals were filtered (band-pass, 5 Hz–1 kHz), amplified (2,000×), sampled at 2 kHz, and stored on a PC for off-line analysis. Signal processing and analysis was carried out offline. The EMG records were rectified and low-pass filtered at 40 Hz (first order Butterworth). The trials were then ensemble-averaged to produce a single record for all situations.

To be qualified as a stretch reflex, EMG activity in a window 22–100 ms after onset of perturbation had to be more than 50 μV above the background EMG. The window 22–100 ms was chosen since the onset latency of the short-latency reflex is around 40 ms in adults and the voluntary reaction time is around 100 ms. The reflex torque was measured as the peak torque in a 360 ms window starting 100 ms after the end of the incline ramp phase of the perturbation relative to the baseline torque at the end of the hold period (Figure 1D; amplitude difference between black arrows). This has previously been shown to give a valid estimate of reflex torque (Toft et al., 1991). Lorentzen et al. (2010) demonstrated that the reflex torque measured in this way was abolished when transmission in large diameter afferents was blocked by ischemia induced by a blood pressure cuff placed around the thigh and inflated to 240 mm Hg (Lorentzen et al., 2010).

Passive torque was the difference between maximum torque during the slow trials (7 deg/s) and the resting torque at each ankle angle (Figure 1C; indicated by black arrows). The mean of the 10 perturbations was calculated (Maganaris et al., 2002; Lorentzen et al., 2010; Willerslev-Olsen et al., 2013).

The torque elicited by a stretch reflex depends on the number of cross-bridges between actin and myosin filaments that may be created. Since this is likely to vary between subjects the reflex torque was normalized to the maximal plantarflexion torque elicited by supramaximal stimulation of the tibial nerve at each of the investigated ankle joint positions. The stimulation was applied to the tibial nerve by a ball electrode (cathode) placed in the popliteal fossa and a metal plate (anode) placed above the patella using an electrical stimulator (Digitimer DS7AH, United Kingdom). The stimulation was a single shock of 1 ms duration at 300 V and up to 1 A. Since this normalization did not change the findings and since a similar normalization would not make sense in relation to the passive torque (since no cross-bridges are formed in the absence of muscle activity), we have chosen to report only non-normalized data.

### Ultrasound

A personal computer-based ultrasound system (LogicScan 128; Telemed, Vilnius, Lithuania) and a 128-element linear probe (B-mode; 7.5 MHz; 60 mm field of view) were used to image the medial gastrocnemius (MG) muscle fascicles at a sampling frequency of 80 Hz (Figure 1B). Depth was set to 60 mm, power and gain were both set to 90%. The ultrasound recordings of the MG muscle were performed during the fast and slow stretch perturbations for 10 s which allowed recording of two fast and two slow stretch perturbations at each angle position (Figures 1C,D). The probe was positioned over the MG and aligned with the fascicle plane (Bénard et al., 2009) to minimize errors attributable to probe orientation (Klimstra et al., 2007) and secured over the skin surface with a compressive bandage to minimize probe movement relative to the skin. A digital output signal from the ultrasound system was used to synchronize data collection of torque, EMG and changes in fascicles. MG muscle fascicle lengths were determined throughout the slow passive and reflex stiffness trials using a semi-automated fascicle tracking algorithm (Cronin et al., 2011; Gillett et al., 2012). The dynamometer measurements of ankle angle and torque, EMG and ultrasound fascicle length data were integrated using customised Matlab (R2015b, The MathWorks, MA, United States) scripts.

Effort was made to keep the test conditions similar for each of the tests. Special focus was on: seating position according to the above mentioned angles; the room temperature (approximately 20°C); no direct, sharp light from windows; that there was quiet in the room while testing, that the subjects had emptied their bladder prior to the experiments, and that the subjects were sitting relaxed during all measurements.

### Clinical Evaluation

On the same day of the biomechanical and electrophysiological test each patient underwent a thorough clinical neurological examination conducted by an experienced physiotherapist specialized in neurology. The neurological examination included evaluation of tonus, ankle ROM, and muscle strength in both lower limbs.

Spasticity was evaluated clinically by use of the Modified Ashworth scale (Bohannon and Smith, 1987) which is a five point ordinal scale from 0 = no increase in tone to 4 = Limb rigid in flexion or extension. The Achilles reflex was evaluated by striking the Achilles tendon with a rubber hammer. The Achilles reflex was categorized as either “normal/no reflex response” or “hyperactive.”

The examinations were used to decide which extremity that was most affected by hypertonia and/or hyperreflexia. The most hypertonic leg was subsequently selected for the further biomechanical electrophysiological test.

The main results from the clinical tests are summarized in Table 1.

All measurements were carried out either in the morning or in the early afternoon with equal proportions for each of the groups. NI adults were matched with regard to the time of measurement for adults with CP.

Parameters of interest included ankle angle (deg), ankle passive- and reflex torque (Nm), MG fascicle length (mm), fascicle length change (mm), and velocity of fascicle length change (mm/s). Fascicle velocities were determined by differentiating fascicle length with respect to time during fast and slow perturbations (Cronin et al., 2013). Fascicle length change for MG was calculated as the difference in MG fascicle length from beginning (baseline) to end of the fast and slow stretch.

### Statistics

All statistical analysis was performed in SPSS 22 and figures were made in Sigmaplot 13.0. MG fascicle length and torque parameters during stretches were analyzed using separate linear mixed model analysis. Significant interaction and main effects of Ankle joint position (70, 80, 90, 100, 110, and 120 deg) and group (CP and NI) were explored. The range 70–120 deg was chosen since a position of 60 deg was not possible in any of the adults with CP and a position of 130 deg was only possible in four adults with CP. A position of 70 and 80 deg was obtained in 7 and 8 of the adults with CP, respectively. It was possible to obtain measurements in all adults with CP for the remaining positions and for all ankle joint positions for NI adults.

With significant main effects *post hoc* pairwise comparisons were performed using one-way ANOVA to detect group differences. Tukey adjustment was used in the *post hoc* comparisons to limit the risk of false positive results.

Correlations between torque and fascicle length measurements were analyzed by Spearman correlation coefficient.

The significance level for all statistical tests was set to 0.05.

## Results

**FIGURE 2:**
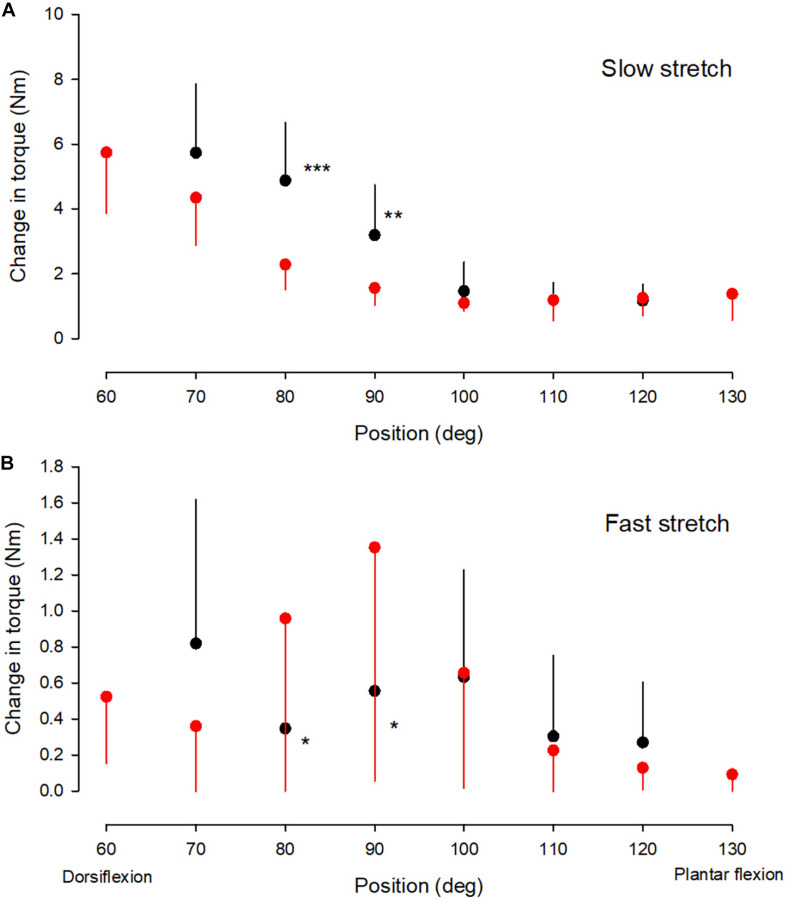
Torque values. The change in passive torque **(A)** and reflex torque **(B)** of the ankle plantarflexor muscle-tendon unit at different ankle joint positions throughout the range of movement in the NI (red circles) and CP group (black circles). It was not possible to obtain measurements at joint positions of 60 and 130 deg in the majority of adults with CP and only measurements from the NI group are therefore shown for these two positions. It was possible to obtain measurements from 7 of the 13 adults with CP at 70 deg and these measurements have therefore also been included. For the remaining positions measurements were obtained from all subjects. Each symbol represents the mean of all measurements in each of the groups at the respective ankle joint positions. Each vertical bar is 1 SD. Statistically significant differences in measurements between the two groups are indicated by stars. *p < 0.05, **p < 0.01, ***p < 0.001.

Mixed model analysis revealed a significant interaction between ankle joint position and group for the amount of passive torque (Figure 2A; *df* = 6; *F* = 7.4; *p* < 0.001), but not for the amount of reflex torque (Figure 2B; *df* = 6; *F* = 1.9; *p* = 0.099). Passive torque increased significantly with increasing dorsiflexion position as a main effect in the two populations (Figure 2A; *df* = 7; *F* = 33.8; *p* < 0.001). *Post hoc* test revealed significantly larger passive torque in the CP than in the NI group at ankle joint positions of 80 and 90 deg (indicated by asterisks in Figure 2A; *p* < 0.001).

Reflex torque varied significantly with ankle joint position in the two populations (Figure 2B, *F* = 9.4; *p* < 0.001). *Post hoc* test revealed significantly larger reflex torque in the NI group than in the CP group at 80 and 90 deg (*p* < 0.05). Normalization of the reflex torque to the maximal torque elicited by supramaximal stimulation of the tibial nerve showed similar results.

**FIGURE 3:**
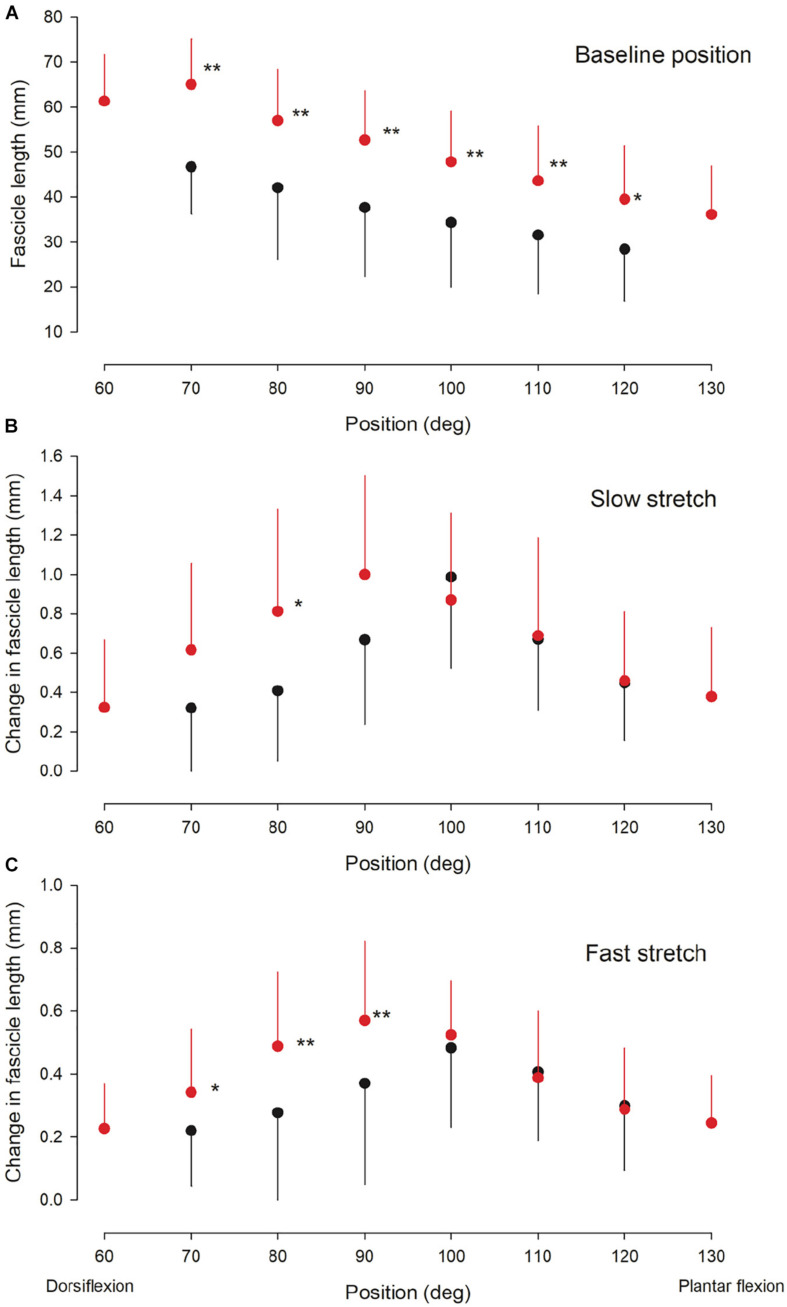
Ultrasound findings. Medial gastrocnemius fascicle length at baseline (rest) **(A)** and the change in medial gastrocnemius muscle fascicle length elicited by a slow **(B)** and fast **(C)** stretch of the ankle plantarflexor muscle-tendon unit at different ankle joint positions throughout the range of movement in the NI (red circles) and CP group (black circles). Otherwise similar legend as for Figure 2. Statistically significant differences in measurements between the two groups are indicated by stars. *p < 0.05, **p < 0.01.

There was no significant interaction between ankle joint position and group for the baseline muscle fascicle length (Figure 3A; *F* = 0.3; *p* = 0.93). However, significantly longer fascicles were observed with increasing dorsiflexion position in the two groups (*df* = 7; *F* = 11.6; *p* < 0.001) and the fascicles were found to be shorter in the CP group than in the NI group (*df* = 1; *F* = 33.3; *p* < 0.001). *Post hoc* test showed that this was the case for all joint positions (Figure 3A; significance level indicated by asterisks). There was no difference in the extent of the change in fascicle length from the most plantarflexed to the most dorsiflexed position (slope) in the two populations (0.36 mm/deg in NI vs 0.37 mm/deg in CP).

There was no significant interaction between ankle joint position and group for the change in fascicle length induced by a slow (Figure 3B; *df* = 6; *F* = 1.6; *p* = 0.16) or fast stretch (Figure 3C; *df* = 6; *F* = 1.3; *p* = 0.28). There was also no significant difference between the two groups for the slow stretch (*df* = 1; *F* = 1.5; *p* = 0.22), whereas a significant difference was found for the fast stretch (*df* = 1; *F* = 4.1; *p* < 0.05). For both types of stretch a significant effect of position was observed (slow stretch: *F* = 5.4; *p* < 0.01; fast stretch: *F* = 4.8; *p* < 0.01). *Post hoc* test showed significantly larger change in fascicle length by the slow stretch in the NI group as compared to the CP group at 80 deg (*p* < 0.05) *Post hoc* test showed significantly larger change in fascicle length with the fast stretch in the NI group as compared to the CP group at ankle joint positions of 70, 80, and 90 deg (*p* < 0.05).

**FIGURE 4:**
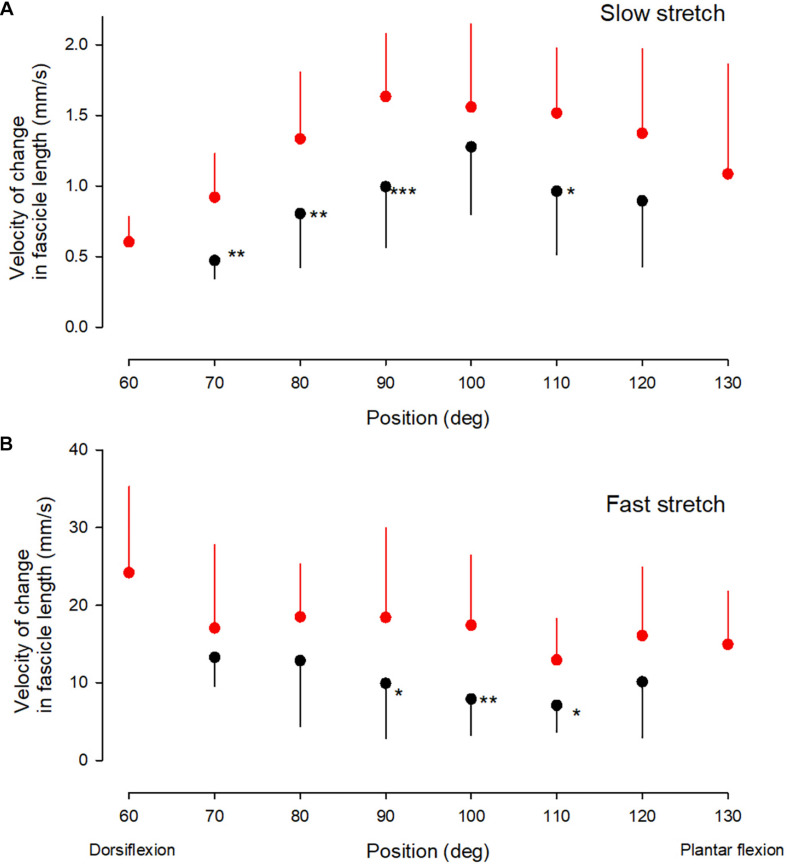
Velocity measurements. Medial gastrocnemius fascicle velocity at different ankle joint positions throughout the range of movement during slow stretch **(A)** and fast stretch **(B)**. Mean, error bar 1 SD. CP, black corcles and NI, red circles. Statistically significant differences in measurements between the two groups are indicated by stars. *p < 0.05, **p < 0.01, ***p < 0.001.

There was no interaction between group and position for the velocity of the change in fascicle length during the slow stretches (Figure 4A; *df* = 1; *F* = 0.38; *p* = 0.85). In both groups the velocity varied significantly with the position of the ankle joint (*df* = 7; *F* = 15.7; *p* < 0.001) with the highest velocities around ankle joint positions of 80–90 deg. Significantly lower velocities were observed across all ankle joint positions in the CP group as compared to the NI group (*df* = 1; *F* = 38; *p* < 0.001). *Post hoc* test showed a significant difference between measurements for the two groups at all ankle joint positions except 100 and 120 deg (marked by asterisks in Figure 4A).

There was no significant interaction between position and group for the velocity of change in fascicle length during the fast stretches (Figure 4B; *df* = 5, *F* = 0.3; *p* = 0.85). The velocity of the change in fascicle length also showed no significant main effect of the position of the ankle joint for the two groups (*df* = 6; *F* = 2.5; *p* = 0.1), but there was a significantly lower velocity in the CP group across all ankle positions (*df* = 1; *F* = 19.5; *p* < 0.001). *Post hoc* test showed a statistically significant difference between measurements in the two groups at ankle joint positions of 90, 100, and 110 deg (marked by asterisks in Figure 4B).

**FIGURE 5:**
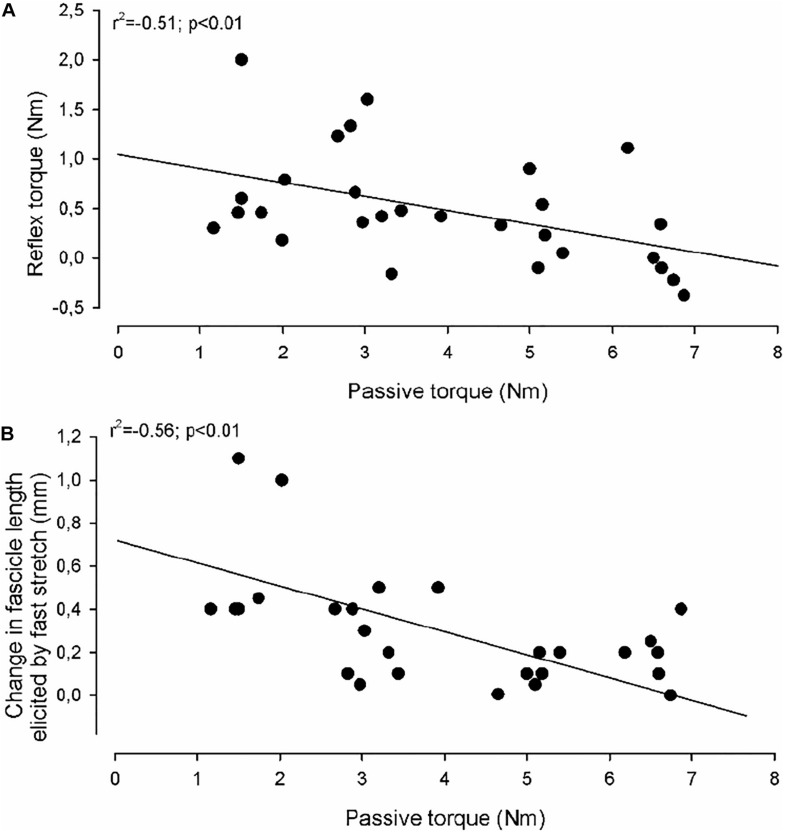
Correlations. In **(A)** the reflex torque in individual adults with CP (elicited by the fast stretch) as a function of the passive torque (elicited by the slow stretch) is shown. **(B)** Shows the change in medial gastrocnemius fascicle length elicited by the fast stretch as a function of the passive torque (elicited by the slow stretch). The full lines indicate the regression lines for the data. The correlation coefficient and statistical significance of the correlation between the measurements using Spearman correlation analysis are given in the upper left part of the graphs.

In the CP group a significant negative correlation was found between the amount of reflex torque and the amount of passive torque at ankle joint positions of 80 and 90 deg (Figure 5A; *r*^2^ = −0.51; *p* < 0.001; data at 80 and 90 deg were pooled together). This was not the case in the NI group or at other ankle joint positions in the CP group (*p* = 0.15–0.75). The amplitude of the fascicle length change imposed by fast stretches at ankle joint positions of 80 and 90 deg was also negatively correlated with the amount of passive torque (Figure 5B; *r*^2^ = −0.56; *p* < 0.01). This was not the case at any other ankle joint positions or in the NI group (*p* = 0.2–0.9). There were also no significant correlations between fascicle length changes and reflex torque in any of the groups (*p* > 0.2). There was also no correlation between the velocity of stretch and the torque measurements at any of the positions or in any of the two groups (*p* > 0.1).

No relation was found between any of the torque measurements and the score on the Modified Ashworth Scale for the adults with CP.

## Discussion

In this study, we have found that passive resistance of the ankle plantar flexor joint increases to a larger extent with increasing dorsiflexion position of the ankle joint in a group of adults with CP than in a group of neurologically intact (NI) adults. At the ankle joint positions where larger passive resistance was observed in the CP population, lower reflex torque was elicited by a fast stretch of the ankle plantar flexor muscle unit in the CP group than in the NI group. At the same ankle joint positions, ultrasound measurements showed that the fast stretch lengthened the muscle fascicles less and at a lower velocity in the CP group than in the NI group. These findings indicate that increased passive resistance of the elastic elements in the ankle plantar flexor muscle-tendon unit may diminish the reflex response to a fast stretch and thereby may conceal exaggerated stretch reflexes in people with spasticity.

All individuals in the group of adults with CP had been found to be spastic at some point in their childhood and all referred to themselves as being spastic. In the neurological examination all showed a score on the Modified Ashworth Scale (MAS) of 1 or above 3 used antispastic medication regularly. The finding that the adults with CP did not show larger reflex torque than NI adults – and even showed lower reflex torque than NI adults around the neutral position of the joint – is at variance with the clinical finding of spasticity. This could not be explained by a difference in muscle volume and strength since similar findings were made when the reflex torque measurements were normalized to the maximal muscle torque elicited by supramaximal nerve stimulation.

A number of other studies have similarly failed to demonstrate exaggerated reflexes using objective biomechanical and electrophysiological techniques in patients, who have been diagnosed clinically with spasticity due to multiple sclerosis (Mirbagheri et al., 2008; Lorentzen et al., 2010; Zhang et al., 2014), stroke (Mirbagheri et al., 2008; Lorentzen et al., 2010), spinal cord injury (Lorentzen et al., 2010), or CP (Yamaguchi et al., 2018). As pointed out in several of these studies one explanation may be that it is difficult as part of the neurological examination to distinguish whether pathologically increased resistance to movement of a joint is caused by exaggerated reflex (neural) activity or (non-neural) changes in the elastic elements in the muscle, tendon, joint and connective tissue around the joint. This is especially a problem for MAS, which is often used to assess and quantify spasticity clinically, but does not allow a distinction between these two components of resistance to joint manipulation (Biering-Sorensen et al., 2006; Alibiglou et al., 2008; Malhotra et al., 2008). In line with this, no correlation with the MAS score and the torque measurements were observed in the study. Notably some of the adults with CP, who showed a score of 3 or more on MAS showed low reflex torque, but high passive torque.

The observation of a negative correlation between the magnitude of the fascicle length change induced by the fast stretch and the passive torque at joint positions of 80 and 90 deg in the CP populations suggests – together with the observation of a similar negative correlation between passive and reflex torque – that increased resistance of the muscle tissue in the CP group may “protect” the muscle spindles from being stretched sufficiently to cause a significant reflex discharge. Similar findings have been reported previously in children with CP (Bar-On et al., 2018). The findings in our study are therefore not simply explained by the known increase of passive stiffness and decrease of reflex stiffness with age (Geertsen et al., 2017).

The protective effect of increased passive stiffness could theoretically explain why reflexes were not hyperexcitable and even reduced as compared to the NI group at these joint positions in the CP group. However, this is difficult to determine with any certainty since we do not know how large the reflexes would have been in the individual subjects if not for the increased passive stiffness. Furthermore, larger reflexes in the CP group were not observed at joint positions where passive torque was comparable in the two groups. Stretch velocity was somewhat lower at all joint positions in the CP group suggesting that the muscle spindles may have been activated less efficiently in the CP group than in the NI group. Although peripheral factors, such as increased resistance of the elastic tissue in the muscle- or surrounding the spindles, are therefore likely to play some role in the lack of reflex hyperexcitability in the adults with CP, other factors should also be considered. One possible contributing factor is that some of the adults with CP were taking regular antispastic medication and although all were asked to stop medication for a 24 h period prior to the measurements, it is possible that some effect of the medication still lingered. It is also a possibility that spasticity has simply disappeared in these adults, since they were originally classified as spastic CP early in their childhood. However, all adults in the CP group were found to score at least 1 on the MAS scale and 5 were identified with hyperactive Achilles reflexes.

Our observation of shorter MG fascicle lengths in the CP group than in the NI group is consistent with previous studies in adults with CP (Barber et al., 2011a; Frisk et al., 2019a). Findings in children and youth with CP have been more variable (Malaiya et al., 2007; Mohagheghi et al., 2008; Barber et al., 2011b; Kruse et al., 2018). Some studies have found shorter fascicle lengths (Mohagheghi et al., 2008; Kruse et al., 2018), whereas others have found similar lengths as in age-matched typically developing peers (Malaiya et al., 2007; Barber et al., 2011b). It is likely that differences in normalization or in group selection explains some of this variability in findings. The more consistent findings in adults with CP may indicate progression of atrophy, passive stiffness and contractures with age in the CP population. A history of inactivity and tendon lengthening surgery over several years may contribute to this. However, longitudinal studies are necessary in order to address this issue.

Slow and fast stretch elicited the largest change in fascicle length at ankle joint positions around 90–100 deg; i.e., around the neutral position of the joint, where the maximal amount of voluntary force may also be generated. At more plantar flexed positions significantly smaller length changes were observed with the stretches in both populations suggesting that the muscle was slack at these positions and that more of the imposed stretch was taken up by the tendon and used to tighten the muscle before the fascicle was stretched (Theis et al., 2016; Kalkman et al., 2018). This likely also explains why no significant reflex torque was elicited by the fast stretch at these positions.

The decrease in the fascicle length change and velocity induced by ankle rotation with more dorsiflexed positions than the neutral position of the joint suggests that more force is required to stretch the fascicles at these positions as also evidenced by the increase in the passive torque imposed by the slow stretch. The decrease in the stretch-induced fascicle length change and velocity was observed at slightly more plantar flexed positions in the CP population (i.e., 100 deg in the CP population vs 90 deg in the NI population) and it was accompanied by significantly larger passive torque at these positions. This likely reflects larger passive stiffness of the elastic tissue in the muscle surrounding the muscle spindles at these positions and is likely related to the presence of contractures in the CP population. This is also evidenced by the fact that measurements at 60 deg dorsiflexion was impossible in the CP population and that measurements were only possible in around 50% of the adults with CP at 70 deg.

The lower reflex torque in the CP adults at 80 and 90 deg cannot be explained by a particular low velocity of the fascicle lengthening at those specific positions, since the fascicle length increased at a lower velocity in the CP group than in the NI group at all positions. There was also no significant correlation between the velocity of the fascicle lengthening imposed by the fast stretches and the evoked reflex torque in any of the two groups regardless of normalization. This is surprising since the reflex torque is assumed to be caused by stretch-velocity dependent muscle spindles (Toft, 1995). Evidently other inter-individual factors influence the size of the reflex torque sufficiently to conceal any relation between stretch velocity and reflex size across the different individuals and joint positions.

### Functional Implications

The muscle characteristics that we have described in adults with CP in the present study are consistent with previous findings of reduced muscle mass, reduced contractile tissue and infiltration of non-contractile tissue leading to increased passive stiffness and contractures (Gillett et al., 2018; Borg et al., 2019; Frisk et al., 2019a). Reduced muscle mass and muscle strength in combination with reduced mobility of the joint due to the increased stiffness and development of contractures appear to be the main determinants of reduced functional capacity and gait ability in these relatively high functioning and ambulant adults with CP (Geertsen et al., 2015; Gillett et al., 2018; Frisk et al., 2019a). Since the natural history of CP alone is unknown, it is unclear whether antispastic treatment, including botulinum toxin, and surgery, have had a negative or positive impact on the functioning of the group. Nevertheless, the negative correlation between fascicle lengthening and passive stiffness as well as reflex torque indicates that altered muscle properties need to be taken into account when evaluating reflex activity in adults with CP. In contrast, the observation that the mechanical response to fast stretch of the plantar flexor muscles was either reduced or similar to NI individuals depending on the ankle joint position, suggests that hyperexcitability of the spinal stretch reflex circuitry is unlikely to be a major functional challenge for voluntary ankle movement in this group of adults with CP. The increased passive stiffness and decreased reflex stiffness at joint angles of 80 and 90 deg in adults with CP suggests that reduced spindle response to stretch may have acted as an adaptive peripheral countermeasure to increased central excitability of the stretch reflex circuitry. The larger peripheral stiffness may help to ensure stability, but at the cost of larger resistance to movement and reduced movement range. It should also be pointed out that reduced muscle spindle sensitivity does not only affect stretch reflex responses, but probably more importantly from a functional point of view also proprioception and the possibility of integrating information from the spindles into central motor commands.

### Clinical Implications

Our findings emphasize the conclusion from several other studies that neural and non-neural components of muscle-tendon stiffness are difficult to tell apart without careful biomechanical and electrophysiological evaluation. Increased resistance of the elastic tissue in the muscles, tendons and joints will require higher and more explosive force in order to stretch the muscles at a sufficiently high velocity to elicit stretch reflex activity and thereby to evaluate the role of reflex hyperexcitability in the clinic. Computer-controlled perturbations using an electromotor as in the present study, guarantees that sufficiently high velocities are reached, but this may not be the case in the clinic where the examiner only have their own ability to generate explosive force to rely on. This emphasizes the need of more precise objective measurement of spasticity in individuals with CP when initially determining the need for anti-spasticity medication and monitoring change in the relative contribution of hyperexcitability and passive resistance from childhood to adulthood (Lorentzen et al., 2012; Bar-On et al., 2014a,b; Yamaguchi et al., 2018).

## Data Availability Statement

The raw data supporting the conclusions of this article will be made available by the authors, without undue reservation.

## Ethics Statement

The studies involving human participants were reviewed and approved by Ethics Committee of the Greater Copenhagen area. The patients/participants provided their written informed consent to participate in this study.

## Author Contributions

All authors participated in the planning and discussion of the study. JL wrote first draft of the manuscript. All authors read, edited, and commented on the manuscript. All authors accepted the final version of the manuscript for publication. All authors contributed to the article and approved the submitted version.

## Conflict of Interest

The authors declare that the research was conducted in the absence of any commercial or financial relationships that could be construed as a potential conflict of interest.

## References

[B1] Alibiglou L. Rymer W. Z. Harvey R. L. Mirbagheri M. M. (2008). The relation between ashworth scores and neuromechanical measurements of spasticity following stroke. *J. Neuroeng. Rehabil.* 5:18. 10.1186/1743-0003-5-18 18627628 PMC2515334

[B2] Barber L. Barrett R. Lichtwark G. (2011a). Passive muscle mechanical properties of the medial gastrocnemius in young adults with spastic cerebral palsy. *J. Biomech.* 44 2496–2500. 21762920 10.1016/j.jbiomech.2011.06.008

[B3] Barber L. Hastings-Ison T. Baker R. Barrett R. Lichtwark G. (2011b). Medial gastrocnemius muscle volume and fascicle length in children aged 2 to 5 years with cerebral palsy. *Dev. Med. Child Neurol.* 53 543–548. 10.1111/j.1469-8749.2011.03913.x 21506995

[B4] Bar-On L. Aertbeliën E. Molenaers G. Dan B. Desloovere K. (2014a). Manually controlled instrumented spasticity assessments: a systematic review of psychometric properties. *Dev. Med. Child Neurol.* 56 932–950. 10.1111/dmcn.12419 24635850

[B5] Bar-On L. Kalkman B. M. Cenni F. Schless S. H. Molenaers G. Maganaris C. N. (2018). The relationship between medial gastrocnemius lengthening properties and stretch reflexes in cerebral palsy. *Front. Pediatr.* 6:259. 10.3389/fped.2018.00259 30338247 PMC6180247

[B6] Bar-On L. Van Campenhout A. Desloovere K. Aertbeliën E. Huenaerts C. Vandendoorent B. (2014b). Is an instrumented spasticity assessment an improvement over clinical spasticity scales in assessing and predicting the response to integrated botulinum toxin type a treatment in children with cerebral palsy? *Arch. Phys. Med. Rehabil.* 95 515–523. 10.1016/j.apmr.2013.08.010 23994052

[B7] Baude M. Nielsen J. B. Gracies J. M. (2019). The neurophysiology of deforming spastic paresis: a revised taxonomy. *Ann. Phys. Rehabil. Med.* 62 426–430. 30500361 10.1016/j.rehab.2018.10.004

[B8] Bax M. Goldstein M. Rosenbaum P. Leviton A. Paneth N. Dan B. (2005). Executive committee for the definition of cerebral palsy. proposed definition and classification of cerebral palsy, April 2005. *Dev. Med. Child Neurol.* 47 571–576. 10.1017/s001216220500112x 16108461

[B9] Bénard M. R. Becher J. G. Harlaar J. Huijing P. A. Jaspers R. T. (2009). Anatomical information is needed in ultrasound imaging of muscle to avoid potentially substantial errors in measurement of muscle geometry. *Muscle Nerve* 39 652–665. 10.1002/mus.21287 19291798

[B10] Biering-Sorensen F. Nielsen J. B. Klinge K. (2006). Spasticity-assessment: a review. *Spinal Cord* 44 708–722. 16636687 10.1038/sj.sc.3101928

[B11] Bodine S. C. (2013). Disuse-induced muscle wasting. *Int. J. Biochem. Cell Biol.* 45 2200–2208. 23800384 10.1016/j.biocel.2013.06.011 PMC3856924

[B12] Bohannon R. W. Smith M. B. (1987). Interrater reliability of a modified ashworth scale of muscle spasticity. *Phys. Ther.* 67 206–207. 10.1093/ptj/67.2.206 3809245

[B13] Borg L. Sporring J. Dam E. B. Dahl V. A. Dyrby T. B. Feidenhans’l R. (2019). Muscle fibre morphology and microarchitecture in cerebral palsy patients obtained by 3D synchrotron X-ray computed tomography. *Comput. Biol. Med.* 107 265–269. 10.1016/j.compbiomed.2019.02.008 30878888

[B14] Bottos M. Feliciangeli A. Sciuto L. Gericke C. Vianello A. (2001). Functional status of adults with cerebral palsy and implications for treatment of children. *Dev. Med. Child Neurol.* 43 516–528. 10.1017/s0012162201000950 11508917

[B15] Carrasco D. I. Vincent J. A. Cope T. C. (2017). Distribution of TTX-sensitive voltage-gated sodium channels in primary sensory endings of mammalian muscle spindles. *J. Neurophysiol.* 117 1690–1701. 28123009 10.1152/jn.00889.2016 PMC5380777

[B16] Cremer N. Hurvitz E. A. Peterson M. D. (2017). Multimorbidity in Middle-Aged adults with cerebral palsy. *Am. J. Med.* 130 744.e9–744.e15. 10.1016/j.amjmed.2016.11.044 PMC5502778 28065772

[B17] Cronin N. J. Avela J. Finni T. Peltonen J. (2013). Differences in contractile behaviour between the soleus and medial gastrocnemius muscles during human walking. *J. Exp. Biol.* 216 909–914. 10.1242/jeb.078196 23197091

[B18] Cronin N. J. Carty C. P. Barrett R. S. Lichtwark G. (2011). Automatic tracking of medial gastrocnemius fascicle length during human locomotion. *J. Appl. Physiol.* 111 1491–1496. 10.1152/japplphysiol.00530.2011 21836045

[B19] de Bruin M. Smeulders M. J. Kreulen M. Huijing P. A. Jaspers R. T. (2014). Intramuscular connective tissue differences in spastic and control muscle: a mechanical and histological study. *PLoS One* 9:e101038. 10.1371/journal.pone.0101038 24977410 PMC4076209

[B20] Frisk R. F. Jensen P. Kirk H. Bouyer L. J. Lorentzen J. Nielsen J. B. (2017). Contribution of sensory feedback to plantar flexor muscle activation during push-off in adults with cerebral palsy. *J. Neurophysiol.* 118 3165–3174. 10.1152/jn.00508.2017 28904105 PMC5814719

[B21] Frisk R. F. Lorentzen J. Nielsen J. B. (2019b). Contribution of corticospinal drive to ankle plantar flexor muscle activation during gait in adults with cerebral palsy. *Exp. Brain Res.* 237 1457–1467. 10.1007/s00221-019-05520-3 30900000

[B22] Frisk R. F. Lorentzen J. Barber L. Nielsen J. B. (2019a). Characterization of torque generating properties of ankle plantar flexor muscles in ambulant adults with cerebral palsy. *Eur. J. Appl. Physiol.* 119 1127–1136. 10.1007/s00421-019-04102-z 30778762

[B23] Geertsen S. S. Kirk H. Lorentzen J. Jorsal M. Johansson C. B. Nielsen J. B. (2015). Impaired gait function in adults with cerebral palsy is associated with reduced rapid force generation and increased passive stiffness. *Clin. Neurophysiol.* 126 2320–2329. 10.1016/j.clinph.2015.02.005 25757398

[B24] Geertsen S. S. Willerslev-Olsen M. Lorentzen J. Nielsen J. B. (2017). Development and aging of human spinal cord circuitries. *J. Neurophysiol.* 118 1133–1140. 10.1152/jn.00103.2017 28566459 PMC5547256

[B25] Gillett J. G. Barrett R. S. Lichtwark G. A. (2012). Reliability and accuracy of an automated tracking algorithm to measure controlled passive and active muscle fascicle length changes from ultrasound. *Comput. Methods Biomech. Biomed. Engin.* 16 678–687. 22235878 10.1080/10255842.2011.633516

[B26] Gillett J. G. Lichtwark G. A. Boyd R. N. Barber L. A. (2018). Functional capacity in adults with cerebral palsy: lower limb muscle strength matters. *Arch. Phys. Med. Rehabil.* 99:900-906.e1. 10.1016/j.apmr.2018.01.020 29438658

[B27] Graham H. K. Rosenbaum P. Paneth N. Dan B. Lin J. P. Damiano D. L. (2016). Cerebral palsy. *Nat. Rev. Dis. Primers* 2:15082. 10.1038/nrdp.2015.82 27188686 PMC9619297

[B28] Jalal N. Gracies J. M. Zidi M. (2020). Mechanical and microstructural changes of skeletal muscle following immobilization and/or stroke. *Biomech. Model Mechanobiol.* 19 61–80. 31280449 10.1007/s10237-019-01196-4

[B29] Kalkman B. M. Bar-On L. Cenni F. Maganaris C. N. Bass A. Holmes G. (2018). Muscle and tendon lengthening behaviour of the medial gastrocnemius during ankle joint rotation in children with cerebral palsy. *Exp. Physiol.* 103 1367–1376. 10.1113/EP087053 30091806

[B30] Klimstra M. Dowling J. Durkin J. L. MacDonald M. (2007). The effect of ultrasound probe orientation on muscle architecture measurement. *J. Electromyogr. Kinesiol.* 17 504–514. 10.1016/j.jelekin.2006.04.011 16919969

[B31] Kruse A. Schranz C. Tilp M. Svehlik M. (2018). Muscle and tendon morphology alterations in children and adolescents with mild forms of spastic cerebral palsy. *BMC Pediatr.* 18:156. 10.1186/s12887-018-1129-4 29743109 PMC5941654

[B32] Lorentzen J. Grey M. J. Crone C. Mazevet D. Biering-Sørensen F. Nielsen J. B. (2010). Distinguishing active from passive components of ankle plantar flexor stiffness in stroke, spinal cord injury and multiple sclerosis. *Clin. Neurophysiol.* 121 1939–1951. 10.1016/j.clinph.2010.02.167 20457538

[B33] Lorentzen J. Grey M. J. Geertsen S. S. Biering-Sørensen F. Brunton K. Gorassini M. (2012). Assessment of a portable device for the quantitative measurement of ankle joint stiffness in spastic individuals. *Clin. Neurophysiol.* 123 1371–1382. 10.1016/j.clinph.2011.11.001 22119175

[B34] Maganaris C. N. Baltzopoulos V. Sargeant A. J. (2002). Repeated contractions alter the geometry of human skeletal muscle. *J. Appl. Physiol.* 93 2089–2094. 12391038 10.1152/japplphysiol.00604.2002

[B35] Malaiya R. McNee A. E. Fry N. R. Eve L. C. Gough M. Shortland A. P. (2007). The morphology of the medial gastrocnemius in typically developing children and children with spastic hemiplegic cerebral palsy. *J. Electromyogr. Kinesiol.* 17 657–663. 10.1016/j.jelekin.2007.02.009 17459729

[B36] Malhotra S. Cousins E. Ward A. Day C. Jones P. Roffe C. (2008). An investigation into the agreement between clinical, biomechanical and neurophysiological measures of spasticity. *Clin. Rehabil.* 22 1105–1115. 10.1177/0269215508095089 19052249

[B37] Mirbagheri M. M. Tsao C. Settle K. Lilaonitkul T. Rymer W. Z. (2008). Time course of changes in neuromuscular properties following stroke. *Annu. Int. Conf. IEEE Eng. Med. Biol. Soc.* 2008 5097–5100. 10.1109/IEMBS.2008.4650360 19163863

[B38] Mohagheghi A. A. Khan T. Meadows T. H. Giannikas K. Baltzopoulos V. Maganaris C. N. (2008). In vivo gastrocnemius muscle fascicle length in children with and without diplegic cerebral palsy. *Dev. Med. Child Neurol.* 50 44–50. 10.1111/j.1469-8749.2007.02008.x 18173630

[B39] Morgan P. McGinley J. (2014). Gait function and decline in adults with cerebral palsy: a systematic review. *Disabil. Rehabil.* 36 1–9. 23594053 10.3109/09638288.2013.775359

[B40] Narici M. Franchi M. Maganaris C. (2016). Muscle structural assembly and functional consequences. *J. Exp. Biol.* 219(Pt. 2), 276–284. 26792340 10.1242/jeb.128017

[B41] Peterson M. D. Ryan J. M. Hurvitz E. A. Mahmoudi E. (2015). Chronic conditions in adults with cerebral palsy. *JAMA* 314 2303–2305. 10.1001/jama.2015.11025 26624831 PMC4862577

[B42] Singer B. Dunne J. Allison G. (2001). Reflex and non-reflex elements of hypertonia in triceps surae muscles following acquired brain injury: implications for rehabilitation. *Disabil. Rehabil.* 23 749–757. 11762877 10.1080/09638280110060466

[B43] Theis N. Mohagheghi A. A. Korff T. (2016). Mechanical and material properties of the plantarflexor muscles and Achilles tendon in children with spastic cerebral palsy and typically developing children. *J. Biomech.* 49 3004–3008. 27515440 10.1016/j.jbiomech.2016.07.020

[B44] Toft E. (1995). Mechanical and electromyographic stretch responses in spastic and healthy subjects. *Acta Neurol. Scand. Suppl.* 163 1–24. 7484084

[B45] Toft E. Sinkjaer T. Andreassen S. Larsen K. (1991). Mechanical and electromyographic responses to stretch of the human ankle extensors. *J. Neurophysiol.* 65 1402–1410. 10.1152/jn.1991.65.6.1402 1875249

[B46] Willerslev-Olsen M. Lorentzen J. Sinkjaer T. Nielsen J. B. (2013). Passive muscle properties are altered in children with cerebral palsy before the age of 3 years and are difficult to distinguish clinically from spasticity. *Dev. Med. Child Neurol.* 55 617–623. 10.1111/dmcn.12124 23517272

[B47] Windhorst U. (2008). Muscle spindles are multi-functional. *Brain Res. Bull.* 75 507–508. 18355625 10.1016/j.brainresbull.2007.11.009

[B48] Yamaguchi T. Hvass Petersen T. Kirk H. Forman C. Svane C. Kofoed-Hansen M. (2018). Spasticity in adults with cerebral palsy and multiple sclerosis measured by objective clinically applicable technique. *Clin. Neurophysiol.* 129 2010–2021. 10.1016/j.clinph.2018.07.004 30053672

[B49] Zhang L. Q. Chen K. Kang S. H. Sliwa J. A. Cohen B. A. Rymer W. Z. (2014). Characterizations of reflex and nonreflex changes in spastic multiple sclerosis. *J. Neurosci. Methods* 231 3–8. 10.1016/j.jneumeth.2014.01.014 24472531

